# Colon Drug Delivery Systems Based on Swellable and Microbially Degradable High-Methoxyl Pectin: Coating Process and In Vitro Performance

**DOI:** 10.3390/pharmaceutics16040508

**Published:** 2024-04-07

**Authors:** Saliha Moutaharrik, Luca Palugan, Matteo Cerea, Gabriele Meroni, Eleonora Casagni, Gabriella Roda, Piera Anna Martino, Andrea Gazzaniga, Alessandra Maroni, Anastasia Foppoli

**Affiliations:** 1 Department of Pharmaceutical Sciences, Section of Pharmaceutical Technology and Legislation “M.E. Sangalli”, University of Milan, Via G. Colombo 71, 20133 Milan, Italy; 2 Department of Biomedical, Surgical and Dental Sciences, One Health Unit, University of Milan, Via Pascal 36, 20133 Milan, Italy; 3 Department of Pharmaceutical Sciences, Section of Medicinal Chemistry “P. Pratesi”, University of Milan, Via Trentacoste 2, 20134 Milan, Italy

**Keywords:** HM pectin, powder-layering, spray-coating, oral colon delivery, small intestinal transit time, colon microbiota

## Abstract

Oral colon delivery systems based on a dual targeting strategy, harnessing time- and microbiota-dependent release mechanisms, were designed in the form of a drug-containing core, a swellable/biodegradable polysaccharide inner layer and a gastroresistant outer film. High-methoxyl pectin was employed as the functional coating polymer and was applied by spray-coating or powder-layering. Stratification of pectin powder required the use of low-viscosity hydroxypropyl methylcellulose in water solution as the binder. These coatings exhibited rough surfaces and higher thicknesses than the spray-coated ones. Using a finer powder fraction improved the process outcome, coating quality and inherent barrier properties in aqueous fluids. Pulsatile release profiles and reproducible lag phases of the pursued duration were obtained from systems manufactured by both techniques. This performance was confirmed by double-coated systems, provided with a Kollicoat^®^ MAE outer film that yielded resistance in the acidic stage of the test. Moreover, HM pectin-based coatings manufactured by powder-layering, tested in the presence of bacteria from a Crohn’s disease patient, showed earlier release, supporting the role of microbial degradation as a triggering mechanism at the target site. The overall results highlighted viable coating options and in vitro release characteristics, sparking new interest in naturally occurring pectin as a coating agent for oral colon delivery.

## 1. Introduction

Oral colon delivery has been extensively investigated to effectively treat intestinal disorders, such as primarily inflammatory bowel disease (IBD), and to replenish the microbiota [1,2,3,4,5]. Furthermore, it is of interest for the administration of biotechnological drugs, namely peptides, proteins, oligonucleotides and nucleic acids, which may face less harsh conditions in the distal gut as compared with the stomach and small intestine due to lower concentrations of digestive enzymes and physiological surfactants [6,7,8,9]. For colon delivery purposes, numerous polysaccharides of natural origin have been exploited [10,11]. These have primarily been incorporated into insoluble or enteric-soluble coatings to trigger release from the core through bacterial degradation once the coated dosage form has entered the large bowel [12,13,14,15,16,17,18,19,20]. Also, they have been used as such to act as matrix-forming or coating agents susceptible to enzymatic breakdown [21,22,23]. Interestingly, such coatings may combine the ability to swell/dissolve/erode upon exposure to aqueous fluids, thus deferring the onset of drug release in the upper gastrointestinal tract, with selective microbial degradability in the colon. The effect of biodegradation would become even more important in case a high amount of polysaccharide has to be applied to prevent possible premature release in the small bowel. This represents a possible advantage over non-biodegradable swellable/erodible polysaccharides used so far according to the time-dependent colon delivery strategy, which may form a persistent swollen polymer layer hindering timely and complete release at the target site [2,24,25]. Such issues have been addressed by incorporating cellulolytic enzymes into a previously described reservoir colon delivery system, either within its hydroxypropyl methylcellulose (HPMC) layer or as an underlying film, with the aim to promote erosion of the functional coating in the colonic region [26].

Among the various polysaccharides used for microbially-triggered colonic release, pectin, a D-galacturonic acid-rich hetero-polysaccharide found in plant cell walls, plays a prominent role [27]. Indeed, it has broadly been used as a colon-targeting excipient, mostly combined with insoluble polymers in an attempt to mitigate the impact of the relevant hydrophilicity and water solubility characteristics and attain a durable barrier [28,29,30,31]. To this end, high-methoxyl pectin has been chosen. Indeed, a higher methoxylation degree reduces solubility and promotes gelation because of lower repulsion forces between free adjacent carboxyl groups [32,33].

Pectin coatings have mainly been applied by press-coating, and only a few attempts to use it as a spray-coating agent have been reported [34,35,36,37,38,39,40]. Nevertheless, press-coating technique is known to involve inherent technical limitations. Particularly, achieving a homogeneous coating thickness has proved challenging, and poor flexibility in the coating level is allowed. In addition, polysaccharides of natural origin may not exhibit acceptable compaction properties [37,41]. Indeed, non-coherent pectin compacts have been described, resulting in powder loss, impaired coating functionality and lack of a consistent release performance.

On the other hand, the main technical hurdles encountered with spray-coating technique are associated with the relatively high viscosity of the aqueous solutions of most natural polysaccharides of interest for colon delivery purposes. This leads to nebulization issues (sprayability), making the use of highly diluted coating formulations and long process times necessary to reach the desired thickness of the applied layer. To overcome such drawbacks, hydro-alcoholic media have been employed, limiting the extent of hydration of the polysaccharides [21,22,42,43]. However, organic solvents are not currently recommended due to safety concerns.

Recently, powder-layering technique has been proposed for the application of time-dependent functional coatings based on HPMC [5,44]. This technique, which has been developed for loading active pharmaceutical ingredients in powder form onto inert cores, consists in the addition of powders and of a binding formulation in alternating or simultaneous mode inside the process chamber of the coating equipment, where the substrates are properly fluidized [45]. The powders adhere to the wet cores, and, due to liquid inter-particle bonds turning into solid structures after solvent evaporation, ultimately form a coherent layer. For HPMC application, the powder-layering process was carried out by a fluid bed apparatus equipped with a tangential-spray insert and compared with aqueous spray-coating using the same equipment in top- and tangential-spray configurations [44]. With powder-layering, the process time was considerably reduced, and the yield was also improved, albeit to a lesser extent. Moreover, such coatings were found to defer the in vitro release as desired, and the relevant performance was comparable or even more efficient than that of spray-coated systems. Based on these results, powder-layering would offer an interesting potential for circumventing major shortcomings of polysaccharide coatings, thus possibly sparking new interest in polymers previously discarded due to processing issues.

Hence, the present work aimed to investigate hydrophilic and microbially degradable high-methoxyl (HM) pectin layers for colon delivery applied either by powder-layering or spray-coating. The coating formulations and process parameters were set up, and physico-technological characteristics as well as in vitro release performance of the coated systems were studied, with a special focus on the relationship between coating technique, structure of the applied layer and relevant efficiency in imparting a lag phase prior to release.

## 2. Materials and Methods

### 2.1. Materials

Paracetamol for direct compression (Rhodapap^TM^ DC 90, Novacyl, Lyon, France) was used as an analytical tracer to study the release performance of the designed formulations. The tablet formulation also included microcrystalline cellulose (Avicel^®^ PH-101, FMC Co., San Colombano al Lambro, Italy), sodium starch glycolate (Explotab^®^ CLV, JRS Pharma, Castenedolo, Italy), vinylpyrrolidone–vinyl acetate copolymer (Kollidon^®^ VA 64, BASF Italia Spa, Cesano Maderno, Italy), hydrophilic fumed silica (Aerosil^®^ 200, Evonik Degussa Italia Spa, Pandino, Italy) and magnesium stearate (Recordati Spa, Milan, Italy). For the coating formulations, high-methoxyl pectin, d10 37.8 µm, d50 135.0 µm, d90 273.0 µm (HM pectin, Aglupectin^®^ HS-RP, JRS Silvateam Ingredients Srl, Bergamo, Italy), hydroxypropyl methylcellulose (HPMC, Methocel^TM^ E5 and Methocel^TM^ K100LV, Colorcon, Dartford, UK), glycerol (ACEF Spa, Fiorenzuola d’Arda, Italy), glycerol monostearate (GMS, Gattefossè SAS, Saint-Priest, France), polysorbate 80 (Tween^®^ 80, ACEF Spa), methacrylic acid and ethylacrylate copolymer (1:1), 30% *w/w* aqueous suspension (Kollicoat^®^ MAE 30DP, BASF Italia Spa), talc (ACEF Spa) and propylene glycol (PG; ACEF Spa) were employed.

### 2.2. Methods

#### 2.2.1. Manufacturing and Characterization of Tablet Cores

A powder mixture having percentage composition as shown in Table 1 was prepared using a bin blender set to 20 rpm (Cyclops, VIMA Impianti Mediterraneo Srl, Castellana Grotte, Italy; 10 + 3 min).

Tablets of 40 mg in nominal weight were obtained by direct compression using a rotary press (AM-8S, Officine Meccaniche F.lli Ronchi, Cinisello Balsamo, Italy) equipped with concave punches (curvature radius and diameter of 4 mm) and characterized according to Ph. Eur. 11 for mass uniformity (analytical balance BP211D Sartorius Italy Srl, Varedo, Italy; *n* = 20), friability (friabilometer TA3R Erweka GmbH, Langen, Germany), crushing strength (crushing tester TBH30 Erweka GmbH; *n* = 10), height and diameter (digimatic indicator Absolute, Mitutoyo Mexicana SA de CV, Naucalpan, Mexico; *n* = 20), disintegration time (three-position disintegration apparatus DT3, Sotax Srl, Milan, Italy; *n* = 6) and dissolution rate (adapted disintegration apparatu, DT3, Sotax Srl, *n* = 3). Before coating, the tablet cores were sealed with an aqueous solution of Methocel^TM^ E5 (5% *w/w*) in a tangential-spray rotary fluid bed (GPCG 1.1, Glatt GmbH, Binzen, Germany). The coating parameters are reported in Section 3. 

#### 2.2.2. HM Pectin Coating

##### Spray-Coating

An aqueous solution of HM pectin was prepared by dispersing the polymer powder in deionized water at a concentration of 1.74% *w/w* under mechanical stirring and heating to 60 °C. A fine dispersion of glycerol monostearate (GMS; 10% on dry polymer mass) was obtained by adding GMS to deionized water and polysorbate (Tween^®^ 80; 40% on dry GMS mass), heating the mixture to 75 °C for 15 min under magnetic stirring and allowing it to cool to room temperature under vigorous stirring [18,19,20]. Glycerol (20% on dry polymer mass) was added to the GMS dispersion before this was mixed with the aqueous HM pectin solution at room temperature under stirring. The resulting formulation was applied onto the tablet cores in a bottom-spray fluid bed (GPCG 1.1, Glatt GmbH) under the coating conditions and up to the coating levels reported in Section 3. The obtained systems were cured in an oven at a temperature of 50 °C for 4 h [40]. 

##### Powder-Layering 

The powder-layering process was carried out in a tangential-spray fluid bed (GPCG 1.1, Glatt GmbH) equipped with a three-way spraying system for powder addition. HM pectin powder, either sieved or not, was fed into the coating chamber at regular time intervals from a hopper. An aqueous solution of Methocel^TM^ E5 (5% *w/w*) or Methocel^TM^ K100LV (3% *w/w*), used as a binder, was nebulized simultaneously or alternately with powder addition. After the process, a final drying phase was carried out in the same apparatus at 60 °C for 10 min. The process conditions and coating levels are reported in Section 3.

##### Kollicoat^®^ MAE Coating

An aqueous dispersion of methacrylic acid and ethyl acrylate copolymer (Kollicoat^®^ MAE 30DP), propylene glycol (PG) and talc at 50, 3 and 4% *w/w*, respectively, was prepared by adding the components to deionized water under magnetic stirring. This dispersion was applied onto tablets provided with pectin-based coatings in a bottom-spray fluid bed (MiniGlatt, Glatt GmbH) up to a nominal 12% weight gain under the process conditions reported in Section 3. 

#### 2.2.3. Characterization of Coated Systems

##### Physico-Technological Characterization

Coated systems were characterized in terms of weight, height and diameter (digimatic indicator Absolute; *n* = 20). The amount of coating materials applied per unit area (*q*), expressed in mg/cm^2^, was calculated as follows:

(1) $$q=\frac{m_{final}-m_{initial}}{A}$$

where *A* is the surface area of the tablet cores, in turn calculated as follows:

(2) $$A\;=\;4\pi \;\left(R-r\right)\;\left(\sqrt{R^{2}-r^{2}}\right)+2\pi rh$$

where *R* and *r* are the curvature radius and radius of the punch, respectively, while *h* is the height of the tablet cores.

The thickness (µm) of the coatings was calculated as the average of the differences between the heights and diameters of coated systems and their respective cores (*n* = 20) and was randomly confirmed by scanning electron microscopy (SEM) analysis. The time equivalent amount parameter (TEAP) was given by the ratio between the amount of coating applied per unit surface (mg/cm^2^) and the in vitro lag time (t_10%_). The time equivalent process parameter (TEPP) was calculated as the process time (min) to in vitro lag time (min) ratio.

The density of the coatings was calculated from the ratio between the amount of coating material applied and the volume of the coating, which was obtained as the difference between the volume of the coated systems and that of the tablet cores. 

Morphology of the coated tablets (cross-sectioned and surface) was analyzed by SEM (JSM-IT500 LA, JEOL, Tokyo, Japan) after gold sputtering (Scancoat SIX, BOC Edwards, Crawley, UK) under vacuum conditions at different magnifications. 

Visual inspection of the systems and monitoring of their macroscopic changes in deionized water at room temperature under static conditions was performed with the aid of photographs taken by a digital microscope (Dyno-Lite Pro AM-413T, AnMo Electronics Co., Hsinchu, Taiwan). 

##### In Vitro Release Test

Release tests (*n* = 3) were carried out by an adapted disintegration apparatus (DT3, Sotax Srl) to avoid sticking of the hydrated polymer layer to the inner wall of the dissolution vessels, which was previously observed and found to impair reliability of results [46]. Different Ph. Eur. 11 fluids (800 mL, 37 ± 1.0 °C) were used, i.e., phosphate buffer (PB) pH 6.8 (0.022 M NaOH and 0.05 M KH_2_PO_4_) or, in the case of double-coated systems, 0.1 N HCl for 2 h and then phosphate buffer pH 6.8. A single dosage form was placed in one tube of the basket-rack assembly, and inlet and outlet pipes were inserted into adjacent tubes. Fluid samples were automatically withdrawn at successive time points, and the drug released was assayed by a spectrophotometer (Lambda 35, PerkinElmer^®^ Italia, Milan, Italy; cuvette 1 mm, λ 248 nm). Lag time (t_10%_) was calculated as the time taken to 10% release in phosphate buffer pH 6.8 by linear interpolation of the closest data before and after this percentage.

Systems with HM pectin-based coating manufactured by powder-layering were also tested in culture medium (1.5 g/L beef extract, 3 g/L yeast extract, 5 g/L tryptone, 2.5 g/L sodium chloride and 0.3 g/L L-cysteine hydrochloride) adjusted to pH 6.5, sterilized in an autoclave (121 °C for 15 min) and enriched with fecal bacteria to give simulated colonic fluid (SCF) according to current fecal microbiota transplantation (FMT) procedures [14,18,19,20,47]. Frozen aliquots (10 mL) of fecal samples from a young Crohn’s disease patient treated with 5-aminosalicylic acid were allowed to thaw at room temperature before each was added to a flask filled with 90 mL of culture medium and incubated under anaerobic conditions and horizontal shaking (50 rpm) at 37 °C for 24 h [20]. The units were transferred to flasks containing 100 mL of either SCF or culture medium as such. The above-described temperature, hydrodynamics and anaerobiosis conditions were maintained throughout the test. At programmed time points, fluid samples (1 mL) were withdrawn, centrifuged at 13,000 rpm for 5 min and filtered (0.22 µm), and the drug released was assayed by HPLC (Nexera-i LC-2040C 3D Plus, Shimadzu, Tokyo, Japan) [18,19,20]. A RAPTOR ARC-18 2.7 µm, 150 × 4.6 mm column (Restek Srl, Cernusco sul Naviglio, Italy) was used, and the mobile phase consisted of (A) orthophosphoric acid 0.085% *v/v* in water and (B) orthophosphoric acid 0.085% *v/v* in acetonitrile. A gradient program was applied as follows: 0–10 min 5–20% B; 10–15 min 20–5% B. Flow rate and injection volume were 1 mL/min and 5 µL, respectively. Paracetamol was detected spectrophotometrically at 250 nm.

## 3. Results and Discussion

The delivery systems were devised in the form of a drug-containing tablet core, a pectin-based inner layer and a gastroresistant outer film to overcome the impact of variable gastric emptying time. 

Powder-layering and spray-coating techniques were employed for application of the inner polysaccharide layer. To compare the outcome of the different techniques employed, pectin was applied to a coating level potentially allowing the drug core to be shielded during small intestinal transit of the dosage form. Particularly, the pectin-based coating was expected to act as a protective barrier for a minimum in vivo time lapse of 4–5 h, according to the colon delivery strategy that relies on small intestinal transit time [2,24,25]. Such a lag time was previously shown to correspond to in vitro lag phases having a duration of 65 to 85 min, as assessed by the release testing procedure in use that resulted in a linear in vitro–in vivo correlation [46].

For the manufacturing of HM pectin-based colon delivery systems, tablets of 4 mm in diameter and nominal weight of 40 mg containing paracetamol as a tracer drug were used as the cores. Their physico-technological characteristics are reported in Table 2.

Prior to spray-coating and powder-layering with pectin formulations, the tablet cores were subjected to sealing to protect them from possible disintegration and mechanical damage during the following steps. Indeed, the aqueous solvent used in the coating processes, even if in a lower amount in powder-layering, and fluidization of the cores inside the coating chamber may have impaired integrity of the substrate. In the specific case of powder-layering, the sealing film applied also aided powder adhesion in the initial coating stages. The sealing phase was conducted in a rotor tangential-spray fluid bed, which reduces mechanical stresses undergone by the cores as compared with the bottom-spray setting of the same equipment. The film was obtained by spraying an aqueous solution of low-viscosity HPMC (Methocel^TM^ E5) up to a weight gain and amount of polymer per unit area of 2.3% and 1.6 mg/cm^2^, respectively, under the operating conditions reported in Table 3. As desired, it was found not to impact the disintegration time (4.10 ± 0.36 min before sealing vs. 4.18 ± 0.90 min after sealing) and dissolution rate (85% release in 4.42 ± 0.10 min before sealing vs. 5.41 ± 0.29 min after sealing) of the drug-containing cores. Subsequently, HM pectin was applied by spray-coating in a bottom-spray fluid bed starting from a 200 g batch of sealed tablets. Preliminary trials showed that the viscosity of aqueous solutions having a pectin percentage higher than 2% *w/w* was excessively high, resulting in sprayability issues. Moreover, to improve the film-forming properties of the polymer and overcome the sticking tendency observed, glycerol at a relatively high concentration and GMS were needed as a plasticizing and an anti-tacking agent, respectively (Table 4) [18,19,20,40]. Spray-coating was carried out under the operating conditions reported in Table 3. Systems having 20, 30, 40 and 50% nominal weight gains were obtained. During the process, interruptions were necessary for cleaning reasons because of nozzle clogging or deposition of coating material on the inner side of the partition cylinder. Finally, the coated systems were heat treated at 50 °C for 4 h, as previously reported [40].

**Figure 1:**
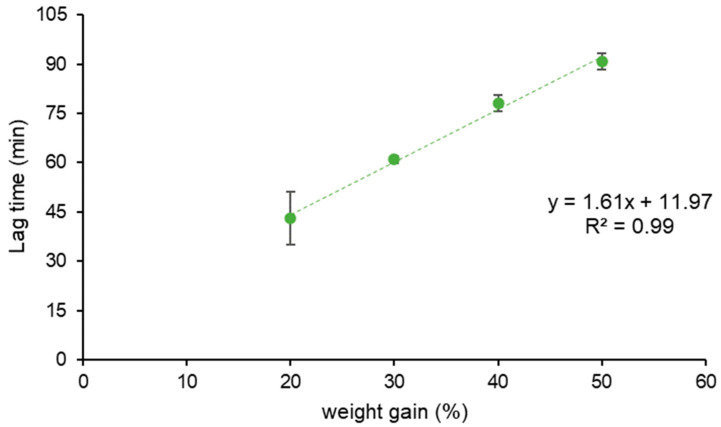
Relationship between in vitro lag time (t_10%_) and weight gain of HM pectin-based systems obtained by spray-coating (bars indicate standard deviation, *n* = 3).

**Figure 2:**
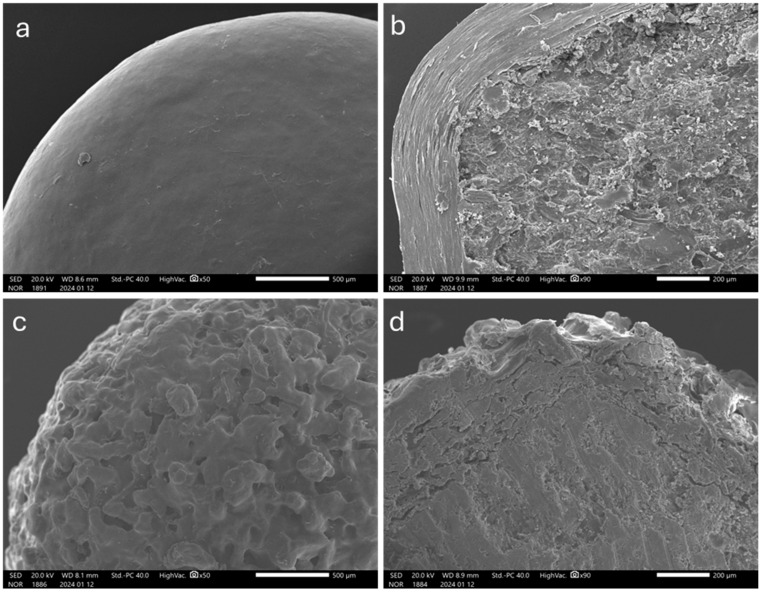
SEM photomicrographs of the surface (**left**) and cross-section (**right**) of HM pectin-based systems having 50% weight gain, obtained by (**a**,**b**) spray-coating and (**c**,**d**) powder-layering (unsieved HM pectin, Methocel^TM^ E5 as the binder).

The obtained systems were tested for release in phosphate buffer pH 6.8, providing typical pulsatile profiles. When t_10%_ (time needed for 10% of the tracer drug to be released) data were plotted vs. the corresponding weight gains, a linear correlation was found (Figure 1). However, a relatively high variability was observed with the lowest coating level, i.e., 20% weight gain. This was ascribed to the limited amount of pectin applied, possibly failing to form a continuous and effective barrier upon hydration. The reproducible release profiles from systems with higher weight gains may indicate the need for a threshold amount of HM pectin for an adequate protection of the drug core to be achieved. The in vitro performance of coated systems having 30 and 50% weight gain, which yielded t_10%_ values of 60.9 ± 1.0 and 90.8 ± 2.4 min, respectively, complied with previously assessed in vitro lag time requirements [46]. Therefore, these weight gains were set as target coating levels. The main physico-technological characteristics of such systems are reported in Table 5. As highlighted by SEM analysis, the applied layer turned out continuous, of consistent thickness and of smooth surface (Figure 2a,b).

**Figure 3:**
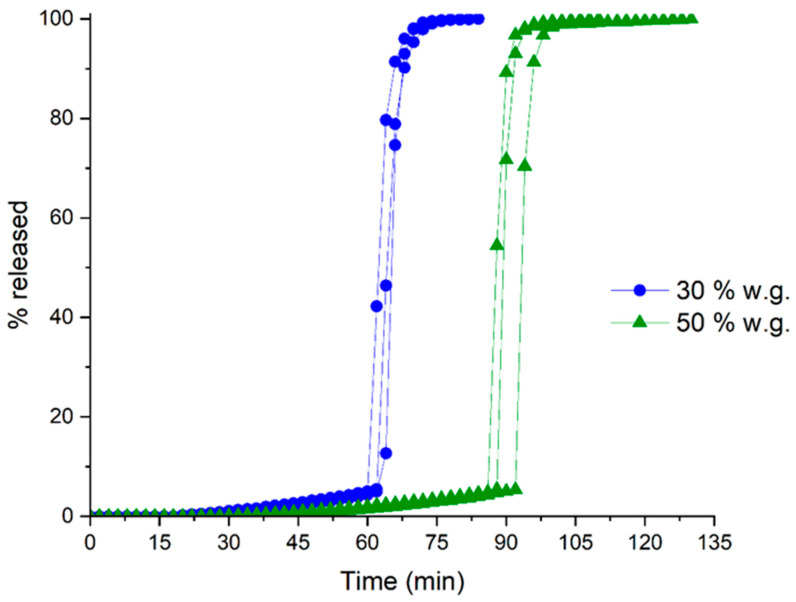
Individual release profiles from HM pectin-based systems obtained by spray-coating with different weight gains.

The release profiles of systems having 30% and 50% weight gain are shown in Figure 3, where drug release appears to be rapid and complete, involving no major diffusional phenomena.

**Figure 4:**
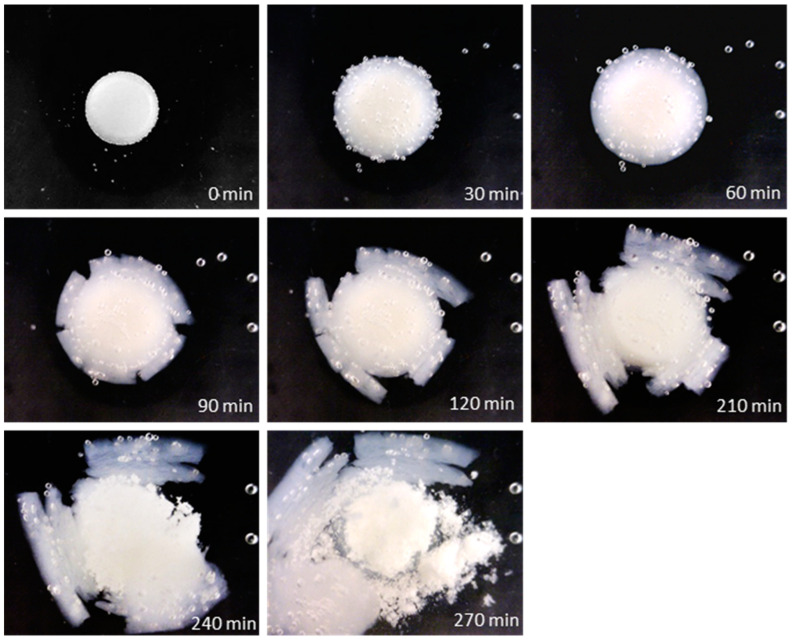
Photographs at successive time points of an HM pectin-based system obtained by spray-coating having 50% weight gain, immersed in unstirred deionized water.

To track hydration and erosion undergone by the pectin-based layer exposed to the aqueous medium, photographs of the coated units immersed in deionized water were taken at successive time points (Figure 4). A progressive detachment of peripheral portions of the gel layer was observed as the polymer swelled. Disintegration of the core occurred only after massive erosion of the hydrated coating.

To circumvent technical issues involved by spray-coating, particularly those resulting from nebulization of viscous aqueous pectin solutions and frequent stops due to nozzle clogging, the use of powder-layering was also explored. Indeed, such a technique was shown to be advantageous in the application of a different swellable/erodible polysaccharide, i.e., low-viscosity HPMC [5,44]. In this case, a tangential-spray fluid bed equipped with a three-way spraying system, which allows the powder and binding solution to be delivered closely, was employed. According to the capacity of the coating chamber of the equipment, a 700 g batch of tablet cores was shown to enable an adequate fluidization pattern both at the beginning of the process and as the weight gain increased.

In initial trials, the stratification of HM pectin powder was attempted using deionized water as the binding liquid to promote adhesion of pectin particles. However, finding an acceptable balance of spray rate and drying air temperature, so that the surface of the tablets would be sufficiently wet for powder deposition without entailing sticking problems, turned out to be challenging. Instead of water as such, an aqueous solution of pectin was therefore investigated as the binder. Even so, layering of the powder on the substrate was still limited in the early stages of the process, and the previously applied sealing began to peel off, pointing out inadequate binding properties of the pectin solution. A 5% *w/w* low-viscosity HPMC (Methocel^TM^ E5) solution was thus employed given its well-known film-forming and adhesion properties as well as reported use in powder-layering [5,44]. The operating conditions were set up to allow for proper motion of the fluidized cores, loading of the coating material and drying of the growing layer (Table 4). The pectin powder and the HPMC solution were added simultaneously into the coating chamber. The binder spray rate was set to a 9.0–13.5 g/min range. The use of HPMC as the binding agent remarkably improved the layering process, promoting effective adhesion of the powder particles onto the cores and then onto the layer in formation. After the desired nominal weight gains of 30% and 50% were reached, a drying phase of 10 min was carried out in the same equipment.

As compared with those manufactured by spray-coating, the resulting systems exhibited a rough surface, which was expected due to the technique in use (Figure 2). Moreover, higher thickness values were obtained for the layered coating, pointing out a lower densification.

**Figure 5:**
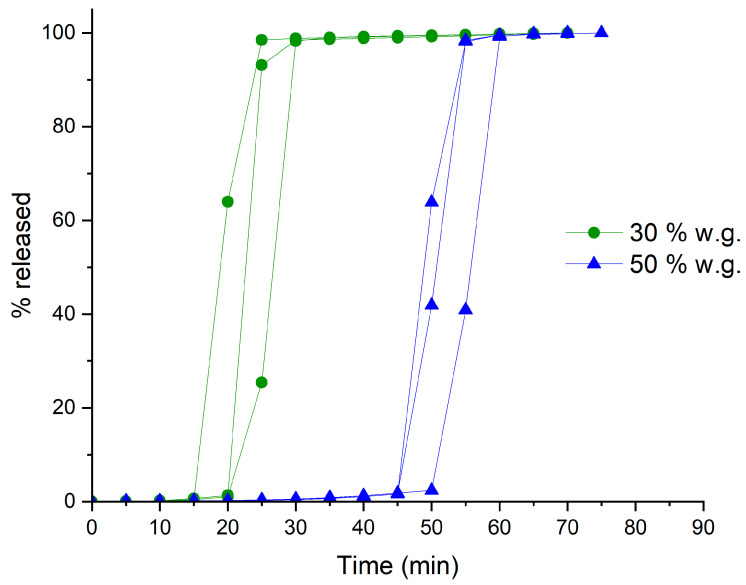
Individual release profiles from HM pectin-based systems having different weight gains, obtained by powder-layering using Methocel^TM^ E5 as the binder.

The release profiles obtained from systems having 30% and 50% nominal weight gains were reproducible, and the drug release was prompt after the lag phase (Figure 5). However, the delay phases were of shorter duration, as expected based on the more porous barriers achievable by powder-layering, and the lag time obtained even from the higher coating level was less than sought.

**Figure 6:**
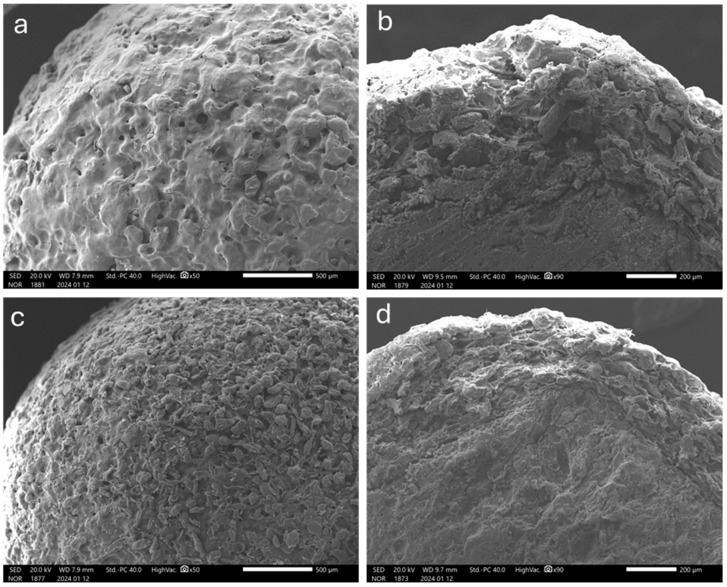
SEM photomicrographs of the surface (**left**) and cross-section (**right**) of HM pectin-based systems having 50% nominal weight gain obtained by powder-layering using Methocel^TM^ K100LV as the binder and (**a**,**b**) unsieved or (**c**,**d**) sieved coating powder.

To extend the lag phases, a higher viscosity grade of HPMC, Methocel^TM^ K100LV, was investigated as the binding agent. In this case, the concentration of HPMC was reduced (3% *w/w*) to rule out possible nebulization issues. The process was carried out under the same coating conditions set before except for the spray rate, which needed to be lowered (6.8–11.3 mg/min) (Table 4). The coated systems were characterized by a rough surface and porous structure as previously observed (Figure 6a,b).

**Figure 7:**
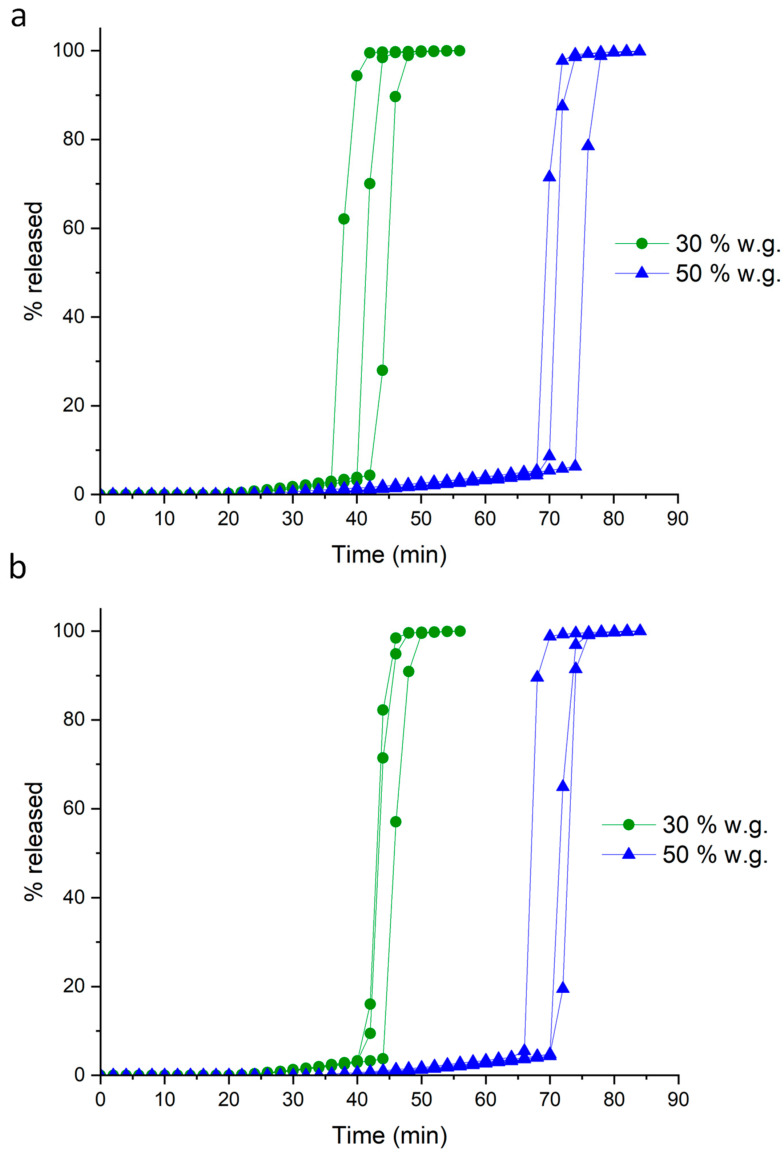
Individual release profiles from HM pectin-based systems having different weight gains, obtained by powder-layering using Methocel^TM^ K100LV as the binder and (**a**) unsieved or (**b**) sieved coating powder.

The use of Methocel^TM^ K100LV as the binder led to an increase in the duration of the in vitro lag phase (Figure 7a). Despite a longer persistence of the hydrated layer, diffusional phenomena before the onset of release were limited. Notably, 50% weight gain was shown to be sufficient to achieve the target lag time.

In addition to a different binder, the possible influence of the particle size of pectin powder was investigated. Indeed, given the peculiar coating technique used, this may have reasonably affected the process outcome, the structure of the applied layer and the in vitro performance in terms of duration as well as reproducibility of the lag phase. Hence, a powder having a narrower size distribution, particularly a sieved fraction of pectin with a <80 µm particle size, was employed. Due to the higher specific surface area of the powder, swelling of the polymer upon exposure to the aqueous binding solution was faster. The consequently increased risk of sticking problems was faced by alternating spraying of the binding solution and feeding of the powder. In this way, it was also possible to prevent the layered powder, having a fine particle size, from being detached and dragged off due to the nebulization pressure, and a more consistent growth of the coating was allowed. Apart from the lower spray rate required (5.1–6.9 g/min), the previously described process conditions were maintained (Table 4).

As can be seen from data in Table 5 and images in Figure 6c,d, the quality of the coating was markedly improved compared to the systems obtained using unsieved pectin. Indeed, the surface was smoother and the thickness values were lower, resulting from a denser structure with closely packed particles. This was reflected in more reproducible in vitro release profiles and reduced diffusional phenomena, although no major differences in the lag phase duration were found (Figure 7b, Table 5).

**Figure 8:**
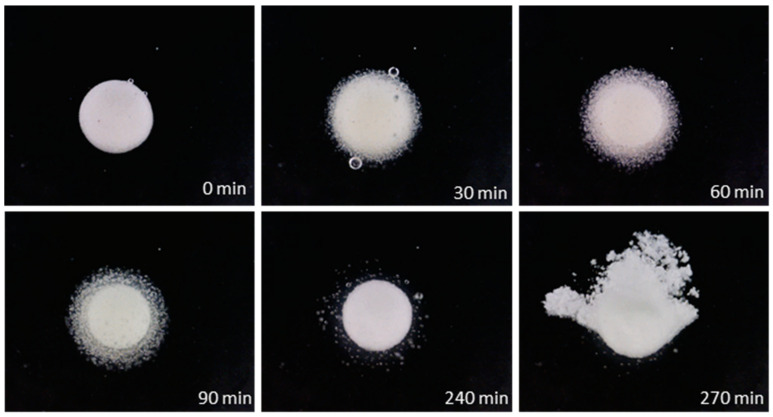
Photographs at successive time points of an HM pectin-based system having 50% weight gain obtained by powder-layering, using sieved pectin and Methocel^TM^ K100LV as the binder, immersed in unstirred deionized water.

Upon exposure of such coated systems to the aqueous medium, a gel layer was formed. However, unlike with spray-coated systems, no bulk erosion was observed (Figure 8). Only after the pectin layer was fully hydrated did the tablet core undergo disintegration. This behavior was ascribed to the different extent of densification related to the employed coating mode.

**Figure 9:**
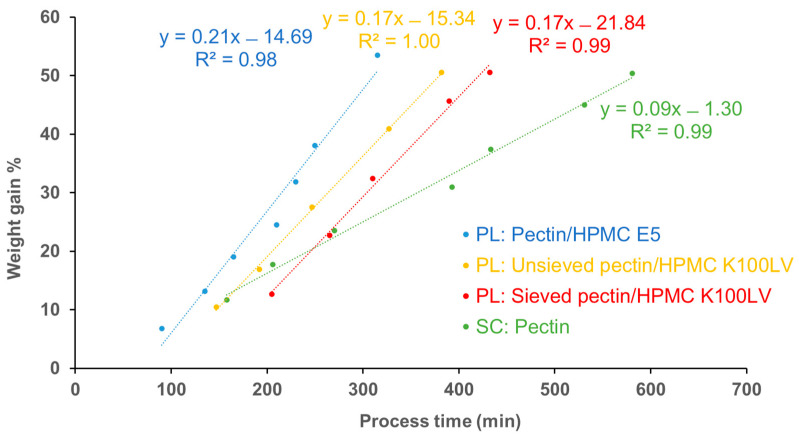
Relationship between weight gain and process time with spray-coating (SC) and powder-layering (PL).

To allow for an overall comparison among the coating processes conducted, weight gains and process times were plotted as shown in Figure 9. Spray-coating generally required a longer processing time than powder-layering. In fact, the use of a highly diluted aqueous solution of pectin in the former case involved long spraying and drying phases, and the interruptions for cleaning operations also had to be accounted for. On the other hand, with powder-layering a relatively low amount of water was used irrespective of the binder employed, thus making more efficient loading of HM pectin possible.

Moreover, the time equivalent amount parameter (TEAP) was calculated as the amount of applied coating material (mg/cm^2^) to in vitro lag time (min) ratio to indicate the coating level required to achieve a 1 min lag phase in vitro (Table 5). Low TEAP values were observed with spray-coated systems, meaning that such coatings had a higher intrinsic release-deferring ability relative to the powder-layering ones. This may result from the different compositions and structural characteristics of the layers obtained by the two techniques. Both included components other than pectin depending on the formula of the sprayed dispersion or binder solution used for each process. In the case of spray-coating (yield 94.4%), equal losses would reasonably occur for all the components, and therefore the percentage of HM pectin in the final layer would be maintained as that of solids in the coating dispersion, i.e., 74.7%. In regard to powder-layering, the percentage loss of pectin and of the HPMC binding solution could be different, and this would be reflected in the composition of the applied layer. If the coating powder and the liquid binder are lost to the same extent, the percentage of HM pectin would be 73.5%, 84.5% and 86.1% in the HM pectin–Methocel^TM^ E5, HM pectin_unsieved_–Methocel^TM^ K100LV and HM pectin_sieved_–Methocel^TM^ K100LV layers, respectively. On the other hand, if the binder loss is negligible with respect to the amount of coating powder lost, the percentages of HM pectin would be 61.9%, 80.6% and 81.7%, respectively, as calculated based on the process yield. Given the relatively high percentage of pectin in the resulting layers (>80% when Methocel^TM^ K100LV was employed as the binder), these would retain their susceptibility to biodegradation by the colon microbiota.

As highlighted by SEM images and confirmed by layer thickness data, the different physical states and deposition modes of the coating material entailed by spray-coating and powder-layering deeply affected the structure of the layers (Figure 2 and Figure 6, Table 5). The relevant density, which is inversely related to porosity, showed a huge difference between spray-coating and powder-layering regardless of the binder and powder particle size employed in the latter process. Only a modest increase in density was observed when finer HM pectin powder in a narrower size distribution range was used as the coating powder. However, such an increase did not appear to affect the in vitro release profiles in terms of extension of the lag phase. Conversely, the impact of density on the in vitro performance was marked with layers applied by spray-coating.

The time equivalent process parameter (TEPP), i.e., the process time (min) to in vitro lag time (min) ratio, indicates the process time required to achieve a 1 min in vitro lag phase. TEPP allows comparison of the efficiency of the processes run by the two techniques under investigation and, in a broader sense, evaluation of the overall advantageousness of coating by either technique. Minor differences were found between HM pectin-coated systems obtained by spray-coating and powder-layering when considering TEPP values relevant to more favorable powder-layering processes using Methocel^TM^ K100LV. The longest process time required by the former technique may indeed be offset by the greater release-deferring ability of the resulting polymer layers, at least in this particular instance. However, it is worth noting that despite a comparable TEPP, powder-layering may allow for a smoother process and a possible finer tuning of the lag phase.

**Figure 10:**
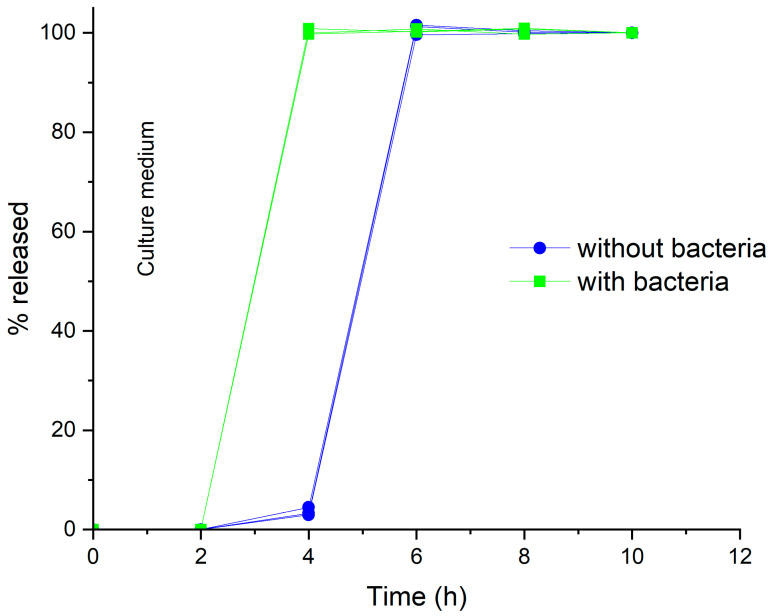
Individual release profiles from double-coated systems with HM pectin-based inner layer (50% weight gain) applied by powder-layering using Methocel^TM^ K100LV as the binder and sieved coating powder, tested in culture medium with (simulated colonic fluid) or without bacteria.

To explore the performance of the HM pectin-based coating in the presence of colonic microbial strains, the system coated to 50% weight gain by powder-layering, using Methocel^TM^ K100LV as the binder and sieved powder, was tested through a purposely designed procedure involving preparation and use of simulated colonic fluid (SCF) with fecal bacteria from an IBD patient. Under such testing conditions, in all cases the lag phases were longer than previously observed, likely due to the different hydrodynamics required. Interestingly, release consistently started 2 h earlier in SCF relative to culture medium without fecal bacteria (Figure 10). This would indicate that the pectin layer could be degraded by the microbiota, thus exerting the hypothesized release-triggering effect in case the swelling/erosion phenomena undergone in the small bowel were not extensive enough to have the drug core exposed by the time of colon arrival.

**Figure 11:**
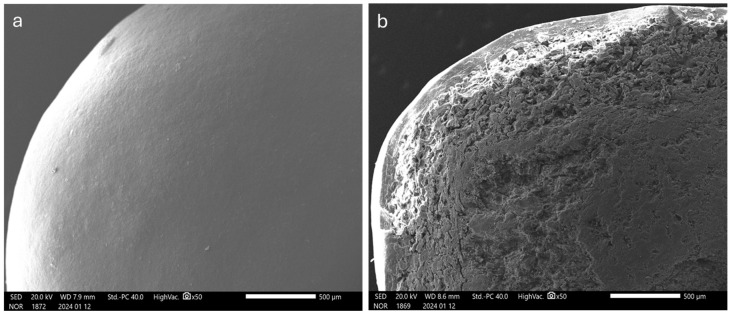
SEM photomicrographs of (**a**) the surface and (**b**) cross-section of systems having an inner layer (50% weight gain) applied by powder-layering, using sieved HM pectin and Methocel^TM^ K100LV as the binder, and a gastroresistant outer film.

**Figure 12:**
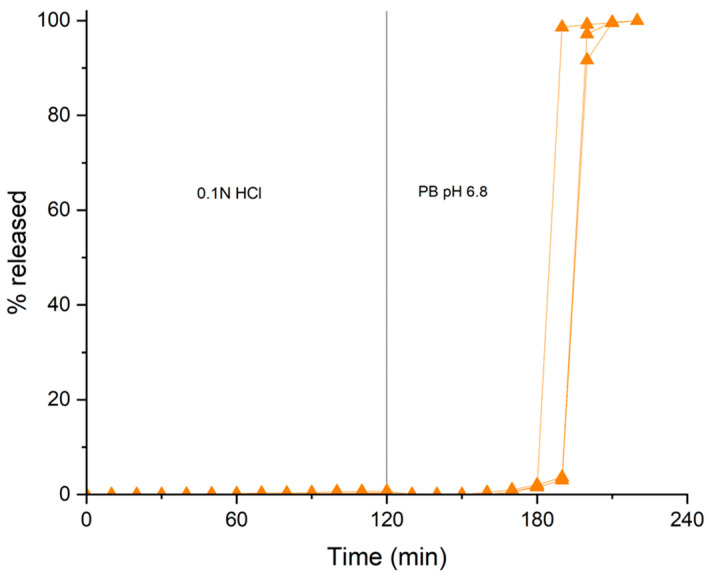
Individual release profiles from double-coated systems with HM pectin-based inner layer (50% weight gain) applied by powder-layering using Methocel^TM^ K100LV as the binder and sieved coating powder.

Finally, the application of a gastroresistant outer film was investigated, which was expected to protect the coated system during variable gastric residence without altering the structural and functional properties of the underlying pectin layer. For the manufacturing of the double-coated colon delivery platform, HM pectin-based systems having 50% weight gain, obtained by powder-layering from the sieved pectin powder fraction and Methocel^TM^ K100LV binding solution, were employed as the coating substrates. These could in principle be more challenging because of the relatively rough surface and low density of the coating, which may allow for penetration of the solvent through the existing pores. Kollicoat^®^ MAE 30DP, an aqueous dispersion of methacrylic acid and ethyl acrylate copolymer soluble at >pH 5.5, was applied in a bottom-spray fluid bed. After setup of the operating conditions, the process, performed with a small-sized batch (50 g), was run with no technical problems apart from few interruptions for nozzle cleaning. The resulting systems were then oven cured for 12 h at 40 °C. A weight gain of 12.8% was reached, corresponding to a thickness of 87.4 µm of the gastroresistant film. SEM analysis showed a smooth surface and homogeneous structure as well as thickness of the outer layer (Figure 11). The double-coated systems withstood 2 h of testing in pH 1.0 hydrochloric acid solution (Figure 12). In phosphate buffer pH 6.8, the pulsatile release pattern, lag phase duration and overall reproducibility of the profiles observed with the non-gastroresistant starting system were maintained. However, the lack of an impact of gastroresistant film dissolution on lag time may not fully be reflected in vivo, where the strength of physiological bicarbonate buffer is known to be lower [48].

## 4. Conclusions

Colon delivery systems based on a dual targeting strategy, involving time- and microbiota-dependent release mechanisms, were proposed. The delivery systems were designed in the form of a drug-containing core, an inner swellable/biodegradable layer and an acrylic gastroresistant outer film. HM pectin, a polysaccharide specifically degraded by the resident microbiota, was employed as the functional coating polymer. So far, pectin has been applied for colon delivery purposes mainly by double-compression, which involves processing and scale-up drawbacks. These limitations, along with the inherent solubility properties impacting its barrier performance, may have shifted interest away from the use of this polymer. In the present work, HM pectin-based layers were thus applied by spray-coating and also by powder-layering, which has only recently been explored for pharmaceutical coating offering major advantages due to the reduced amount of solvent to be removed. Systems manufactured by both techniques generally showed satisfactory physico-technological characteristics and the desired pulsatile release profiles. Although a protective barrier with a higher intrinsic delaying ability was achieved by spray-coating, the process was more time-consuming and less straightforward due to the cleaning interventions required. The coating attained by powder-layering proved susceptible to microbial degradation, and the effectiveness of pectin breakdown in triggering release was assessed in the presence of fecal bacterial strains cultured from an IBD patient.

To manufacture the final double-coated colon delivery platform, the HM pectin-based system obtained by powder-layering from sieved pectin and Methocel^TM^ K100LV was provided with a Kollicoat^®^ MAE gastroresistant film. In vitro results were satisfactory in terms of protection in acidic medium and maintenance of the original release performance after pH change. These findings may spark new interest in naturally occurring and biodegradable pectin for oral colon delivery, considering the highlighted coating process options, the in vitro release characteristics adaptable to the in vivo target and the dual role it may play in the small and large bowel, respectively.

## Figures and Tables

**Table 1:** Composition of tablet cores.

	*% w/w*
Paracetamol DC	80.0
Avicel^®^ PH-101	12.5
Explotab^®^ CLV	4.5
Kollidon^®^ VA 64	2.0
Aerosil^®^ 200	0.5
Magnesium stearate	0.5

**Table 2:** Physico-technological characteristics of tablet cores.

Weight (mg)	40.8 ± 1.2
Height (mm)	3.20 ± 0.05
Diameter (mm)	4.00 ± 0.01
Crushing strength (N)	47.1 ± 7.3
Friability (%)	0.23
Disintegration time (min)	4.10 ± 0.36

**Table 3:** Equipment and operating conditions used for HPMC, pectin and Kollicoat^®^ MAE coating processes.

	HPMC Seal-Coating	Pectin Spray-Coating	Pectin Powder-Layering	Kollicoat^®^ MAE Spray-Coating
Equipment	Tangential-spray fluid bed	Bottom-spray fluid bed	Tangential-spray fluid bed	Bottom-spray fluid bed
Inlet air temperature (°C)	60	60	60	60
Outlet air temperature (°C)	30–34	40–42	30–32	-
Product temperature (°C)	32–34	38–42	30–32	37–39
Disk rotation speed (rpm)	400	-	400	-
Nebulization air pressure (bar)	2	2	2	1.0
Nozzle port diameter (mm)	1.2	0.8	1.2	0.5
Inlet air volume (m^3^/h)	60	60	60	34–40
Spray rate (g/min)	11.2–11.9	8.5	5.1–13.5	1–1.3
Powder feeding rate (g/min)	-	-	2	-

**Table 4:** HM pectin formulation used for spray-coating.

	*% w/w*
HM pectin	1.74
Glycerol	0.35
GMS	0.17
Tween^®^ 80	0.07
Deionized water	97.67

**Table 5:** Physico-technological characteristics of HM pectin-based systems obtained by spray-coating and powder-layering.

Coating Technique	Composition	Weight Gain (%)	Amount of Coating Material Applied (mg/cm^2^)	Coating Thickness (µm)	t_10%_ Mean ± SD (min)	Process Time (min)	TEAP mg/cm^2^·min	Coating Density g/mL	TEPP
Spray-coating	HM pectin	28.3	23.6	148.9	60.9 ± 1.0	393	0.39	1.46	6.45
		47.0	39.1	242.0	90.8 ± 2.4	581	0.43	1.42	6.40
Powder-layering	HM pectin–Methocel^TM^ E5	31.4	24.9	279.5	19.4 ± 3.2	230	1.28	0.77	11.80
		53.5	42.3	438.5	47.6 ± 3.0	315	0.89	0.77	6.62
	HM pectin_unsieved_–Methocel^TM^ K100LV	30.5	24.3	312.9	39.6 ± 3.2	247	0.64	0.67	6.24
		50.5	40.3	457.2	70.8 ± 3.0	382	0.58	0.71	5.39
	HM pectin_sieved_–Methocel^TM^ K100LV	32.5	25.4	251.8	42.4 ± 1.6	272	0.57	0.89	6.41
		50.6	40.9	385.1	69.0 ± 2.5	432	0.58	0.87	6.26

## Data Availability

Data are contained within the article.
